# Correction: A β-catenin-driven switch in TCF/LEF transcription factor binding to DNA target sites promotes commitment of mammalian nephron progenitor cells

**DOI:** 10.7554/eLife.69853

**Published:** 2021-04-29

**Authors:** Qiuyu Guo, Albert D Kim, Bin Li, Andrew Ransick, Helena Bugacov, Xi Chen, Nils O Lindström, Aaron Brown, Leif Oxburgh, Bing Ren, Andrew P McMahon

Guo Q, Kim A, Li B, Ransick A, Bugacov H, Chen X, Lindström N, Brown A, Oxburgh L, Ren B, McMahon AP. 2021. A β-catenin-driven switch in TCF/LEF transcription factor binding to DNA target sites promotes commitment of mammalian nephron progenitor cells. *eLife* **10**:e64444. doi: 10.7554/eLife.64444.

Published 15, February 2021

We overlooked an important published study by Ramalingam et al (2018) which draws the conclusion that different levels of ß-catenin, resulting from different levels of Wnt9b ligand, determine nephron progenitor cell (NPC) outcomes. Though our study focuses on defined NPC cultures and genomic interactions amongst ß-catenin and Tcf/Lef transcriptional mediators that were not explored in Ramalingham et al., our approaches agree on the importance of ß-catenin levels in modifying the state of NPCs. Accordingly, we have corrected our manuscript below to acknowledge Ramalingam et al (2018) were appropriate and we apologize to the authors for the oversight.

#1

Original text:

Genomic analysis of β-catenin engagement at TCF/LEF recognition motifs within enhancers linked to genes driving NPC differentiation (Park et al., 2012), and subsequent transgenic studies demonstrating TCF/LEF-dependent activity of cis regulatory elements, provides strong evidence for a canonical Wnt/β-catenin/Tcf regulatory axis (Mosimann et al., 2009).

Corrected text:

Genetic and chemical modification of Wnt pathway components suggest different levels of β-catenin distinguish maintenance and commitment of NPCs (Ramalingam et al., 2018). Genomic analysis of β-catenin engagement at TCF/LEF recognition motifs within enhancers linked to genes driving NPC differentiation (Park et al., 2012), and subsequent transgenic studies demonstrating TCF/LEF-dependent activity of cis regulatory elements, provides strong evidence for a canonical Wnt/β-catenin/Tcf regulatory axis (Mosimann et al., 2009).

#2

Original text:

Within 3 days of elevating CHIR levels (3 µM), aggregate NPC cultures show a robust signature of nephron differentiation (Brown et al., 2015)

Corrected text:

Within 3 days of elevating CHIR levels (3 µM), aggregate NPC cultures show a robust signature of nephron differentiation (Brown et al., 2015; Ramalingam et al., 2018)

#3

Original text:

Genetic analysis of mouse models points to a requirement for β-catenin in both the maintenance (Karner et al., 2011) and differentiation (Park et al., 2007) of NPCs. Previous studies directly examining β-catenin association in differentiating NPCs showed direct engagement of β-catenin at enhancers regulating expression of differentiation promoting genes such as *Wnt4* (Park et al., 2012)

Corrected text:

Genetic analysis of mouse models points to a requirement for β-catenin in both the maintenance (Karner et al., 2011) and differentiation (Park et al., 2007) of NPCs. Genetically modulating Wnt inputs and chemically modifying Wnt-pathway activity suggest Wnt-modulation of β-catenin levels is critical to these alternative outcomes (Ramalingam et al., 2018). Previous studies directly examining β-catenin association in differentiating NPCs showed direct engagement of β-catenin at enhancers regulating expression of differentiation promoting genes such as *Wnt4* (Park et al., 2012).

#4

Insert reference below into reference list:

**Ramalingam H**, **Fessler AR**, **Das A**, **Valerius MT**, **Basta J**, **Robbins L**, **Brown AC**, **Oxburgh L**, **McMahon AP**, **Rauchman M and Carroll TJ**. (**2018**). **Disparate levels of beta-catenin activity determine nephron progenitor cell fate**. ***Developmental Biology****440*(1)**:13-21**.

#5 (an independent typographic error)

Original text:

*Sub section title* - Elevating β-catenin leads to activation of Tcftt/Lef-bound enhancers

Corrected text:

*Sub-section title* - Elevating β-catenin leads to activation of Tcf/Lef-bound enhancers

